# Bradykinin Induces TRPV1 Exocytotic Recruitment in Peptidergic Nociceptors

**DOI:** 10.3389/fphar.2016.00178

**Published:** 2016-06-23

**Authors:** Sakthikumar Mathivanan, Isabel Devesa, Jean-Pierre Changeux, Antonio Ferrer-Montiel

**Affiliations:** ^1^Instituto de Biología Molecular y Celular, Universitas Miguel HernándezElche, Spain; ^2^College de FranceParis, France; ^3^Centre Nationale de la Recherche Scientifique, Institute Pasteur, Unité de Recherche AssociéeParis, France

**Keywords:** nociceptors, TRPV1, inflammation, neuropeptides, exocytosis

## Abstract

Transient receptor potential vanilloid I (TRPV1) sensitization in peripheral nociceptors is a prominent phenomenon that occurs in inflammatory pain conditions. Pro-algesic agents can potentiate TRPV1 activity in nociceptors through both stimulation of its channel gating and mobilization of channels to the neuronal surface in a context dependent manner. A recent study reported that ATP-induced TRPV1 sensitization in peptidergic nociceptors involves the exocytotic release of channels trafficked by large dense core vesicles (LDCVs) that cargo alpha-calcitonin gene related peptide alpha (αCGRP). We hypothesized that, similar to ATP, bradykinin may also use different mechanisms to sensitize TRPV1 channels in peptidergic and non-peptidergic nociceptors. We found that bradykinin notably enhances the excitability of peptidergic nociceptors, and sensitizes TRPV1, primarily through the bradykinin receptor 2 pathway. Notably, bradykinin sensitization of TRPV1 in peptidergic nociceptors was significantly blocked by inhibiting Ca^2+^-dependent neuronal exocytosis. In addition, silencing αCGRP gene expression, but not substance P, drastically reduced bradykinin-induced TRPV1 sensitization in peptidergic nociceptors. Taken together, these findings indicate that bradykinin-induced sensitization of TRPV1 in peptidergic nociceptors is partially mediated by the exocytotic mobilization of new channels trafficked by αCGRP-loaded LDCVs to the neuronal membrane. Our findings further imply a central role of αCGRP peptidergic nociceptors in peripheral algesic sensitization, and substantiate that inhibition of LDCVs exocytosis is a valuable therapeutic strategy to treat pain, as it concurrently reduces the release of pro-inflammatory peptides and the membrane recruitment of thermoTRP channels.

## Introduction

Transient receptor potential vanilloid I (TRPV1) is a non-selective cation channel that can be activated by noxious heat (T ≥ 42°), vanilloids, protons, voltage, toxins, and membrane derived lipids (Caterina et al., 1997; Zygmunt et al., 1999; Huang et al., 2002; Chu et al., 2003). Physiologically, TRPV1 acts as a major integrator of painful stimuli in nociceptors. During inflammation, the release of inflammatory mediators potentiate TRPV1 activity leading to enhanced nociceptor excitability that results in thermal hyperalgesia (Planells-Cases et al., 2005; Huang et al., 2006; Siemens et al., 2006; Yu et al., 2008). In rats, TRPV1 is widely expressed in both medium size Aδ-fibers and small size C-fibers including peptidergic and non-peptidergic subpopulations (Mitchell et al., 2010). At variance with rats, the expression of TRPV1 is primarily restricted to the peptidergic subpopulation in adult mice (Cavanaugh et al., 2011). Cumulative evidence indicates that acute inflammatory sensitization of TRPV1 involves both the modification of channel gating properties by phosphorylation (Srinivasan et al., 2008) and the recruitment of channels to the neuronal surface (Morenilla-Palao et al., 2004). Mobilization of new channels to the plasma membrane induced by some pro-inflammatory mediators occurs through SNARE-dependent exocytosis (Camprubí-Robles et al., 2009), but the exact mechanism involved remains under investigation. A recent study showed that ATP-induced inflammatory recruitment of TRPV1 channels occurs specifically in peptidergic nociceptors by Ca^2+^-dependent exocytosis of large dense core vesicles (LDCVs; Devesa et al., 2014). Furthermore, evaluation of the cellular mechanism revealed that the neuropeptide αCGRP was essential for the inflammatory recruitment of TRPV1 channels in peptidergic nociceptors. This finding suggests that a similar mechanism may underlie the potentiation of TRPV1 by other pro-inflammatory agents. In support of this tenet, inhibition of TRPV1 recruitment by peptide DD04107, a blocker of neuronal exocytosis (Camprubí-Robles et al., 2009), exhibits notable anti-nociceptive activity in models of inflammatory and neuropathic pain (Ponsati et al., 2012).

Bradykinin (BK) is a potent algogenic non-apeptide released from mast cells and basophils during tissue damage (Dray and Perkins, 1993; Raidoo and Bhoola, 1998; Cesare et al., 1999). BK exerts its inflammatory action through BKR1 and BKR2 metabotropic receptors, which trigger second messenger signaling through Gαq proteins (Mizumura et al., 2009). BKR2 receptor expression is ubiquitous and constitutive, whereas, BKR1 receptor is expressed under chronic inflammatory conditions. It has been documented that BK can sensitize TRPV1 through PKC and PKA-mediated phosphorylation of specific intracellular Ser/Thr sites in the receptor, leading to enhanced activity (Cesare and McNaughton, 1996; Stucky and Lewin, 1999; Premkumar and Ahern, 2000; Sugiura et al., 2002; Zhang et al., 2008; Wang et al., 2015), and that this sensitization may not involve recruitment of new channels to the plasma membrane (Camprubí-Robles et al., 2009). However, numerous *in vitro* studies on primary cultures of nociceptors have reported a possible BK-induced stimulation of neuronal trafficking of receptors to the cell surface. In this regard, BK-induced trafficking of opioid receptors in nociceptors has been studied (Patwardhan et al., 2005; Pettinger et al., 2013). Likewise, BK has also been involved in the Ca^2+^-dependent exocytotic release of αCGRP in primary sensory neurons (Meng et al., 2007; Supowit et al., 2011), as BK releases Ca^2+^ from the endoplasmic reticulum. These evidences suggest that BK may also potentiate TRPV1 activity in nociceptors by promoting its membrane mobilization in peptidergic nociceptors akin to ATP (Devesa et al., 2014).

Here, we investigated this hypothesis and studied the mechanism of BK-induced inflammatory sensitization of TRPV1 in cultured peptidergic and non-peptidergic nociceptors. We found that inhibition of neuronal exocytosis with DD04107 resulted in a decreased BK-induced sensitization of TRPV1 in peptidergic nociceptors but not in non-peptidergic sensory neurons. In addition, knocking out αCGRP expression, but not substance P, markedly reduced BK-evoked potentiation of TRPV1 in peptidergic nociceptors. Hence, our findings substantiate an essential role of neuronal exocytosis of LDCVs containing αCGRP in the potentiation of TRPV1 by pro-algesic agents in peptidergic C-type nociceptors.

## Materials and Methods

### Chemicals

DD04107 (Palmitoyl-EEQMRR-NH_2_), DD04107^RDM^ (Palmitoyl-EQREMR-NH_2_) and non-palmitoylated DD04107 (Ac-EEQMRR-NH_2_) were kindly provided by BCN Peptides, Barcelona, and freshly prepared at 20 mM in H_2_O. For microelectrode array, cells were incubated with 20 μM DD04107 in HBSS for 1 h (37°C, 5% CO_2_). For electrophysiological recordings, non-palmitoylated DD04107 was prepared at 100 μM in standard internal solution from 10 mM stock solution in H_2_O. Peptide or vehicle were applied through the patch pipette 10 min before recording. Capsaicin was dissolved in DMSO at 10 mM and further diluted in HBSS at 500 nM for MEAs and 1 μM for patch clamp experiments. BK was dissolved in H_2_O at 1 mM and further diluted in HBSS at 1 μM. The BKR1 agonist Sar-[D-Phe^8^]-des-Arg^9^-BK and the BKR2 agonist [Phe^8^ψ(CH-NH)-Arg^9^]-BK (Tocris Bioscience, R&D Systems) were dissolved in H_2_O at 1 mM and further diluted in HBSS at 1 μM. The BKR1 antagonist R715 and the BKR2 antagonist HOE140 (Tocris Bioscience, R&D Systems) were dissolved in H_2_O at 1 mM and further diluted in HBSS at 1 μM. Unless indicated, all chemicals were obtained from Sigma-Aldrich.

### Animals

All procedures were approved by the Institutional Animal and Ethical Committee of the University Miguel Hernández de Elche, in accordance with the guidelines of the Economic European Community, the National Institutes of Health, and the Committee for Research and Ethical Issues of the International Association for the Study of Pain. Animals were kept in a controlled environment (21–23°C, 12 h light/dark cycle), and had food and water available *ad libitum*. Neonatal Wistar rats and wild type C57BL/6J mice were purchased from in house bred stock (originally from Harlan Laboratories). Tac1-deficient mice (B6.Cg-Tac1^tm1Bbm^/J) that do not express Substance P were purchased from The Jackson Laboratory. α-CGRP-deficient mice (B6;129-Calca^TM^) were produced as described (Salmon et al., 1999).

### Primary Culture of Sensory Neurons

DRG from neonatal Wistar rats (3–5 days-old) or adult male 12–15 weeks-aged mice (strains: C57BL/6J, αCGRP^-/-^, Tac1^-/-^), were cultured following previously described protocols with some modification (Baker and Bostock, 1997; Bonnington and McNaughton, 2003; Devesa et al., 2014). Briefly, neonatal rat ganglia were digested with 0.25% (w/v) collagenase (type IA) in DMEM-glutamax (Invitrogen) with 1% penicillin-streptomycin (P/S; 5000 U/mL, Invitrogen) for 1 h (37°C, 5% CO_2_). Isolated mouse DRG were incubated with 0.67% (w/v) collagenase type XI and 3% (w/v) dispase (Gibco) in INC mix medium (in mM): 155 NaCl, 1.5 K_2_HPO_4_, 5.6 HEPES, 4.8 NaHEPES, 5 glucose) for 1 h (37°C, 5% CO_2_). After digestion, rat and mouse DRGs were mechanically dissociated using a glass Pasteur pipette. Single cell suspension was passed through a 100 μm cell strainer, and washed with DMEM glutamax plus 10% fetal bovine serum (FBS; Invitrogen) and 1% penicillin/streptomycin. Cells were seeded at the required density for each experiment on 12 mm cover-glass slides, or microelectrode array chambers previously coated with poly-L-lysine (8.3 μg/mL) and laminin (5 μg/mL). After 2 h, medium was replaced with DMEM glutamax, 10% FBS and 1% P/S, supplemented with mouse 2.5S NGF 50 ng/mL (Promega) and 1.25 μg/mL cytosine arabinoside when required (37°C, 5% CO_2_). Unless otherwise indicated, all experiments were made 48 h after cell seeding.

### Patch-Clamp Recordings

Whole-cell voltage clamp was made in neurons seeded on coverslips, placed in RC-25 chamber and connected to a external perfusion system at ≈22°C. External solution was (in mM): 140 NaCl, 4 KCl, 2 CaCl_2_, 2 MgCl_2_, 10 HEPES, 5 D-glucose, 20 mannitol, pH 7.4 (adjusted with NaOH). Internal pipette solution was (in mM): 144 KCl, 2 MgCl_2_, 10 HEPES, 5 EGTA, pH 7.2 (adjusted with KOH). Membrane currents were acquired using EPC10 HEKA Patch amplifier (HEKA Electronics). Cells were held at a RMP of -60 mV, and at a sampling rate of 2.5 Hz. Patch glass pipettes with OD 1.5 mm × I.D. 1.17 mm (Harvard Instruments) were pulled with a Sutter Pippete puller Equipment (Sutter Instruments) to have a 3–6 MΩ resistance. Data acquisition and oﬄine analysis were performed with PatchMaster software (HEKA Electronics). Rat DRG neurons were labeled with IB4-alexa 568 (10 μg/mL, 10 min, RT) in the external solution (Molecular Probes, Invitrogen), followed by two 5 min-washes. Labeled neurons were visualized through x20 air objective (Axiovert 200 inverted microscope, Carl Zeiss), with an excitation filter ET545 and an emision filter ET605 (CHR-49004, Laser 2000 SAD). Neuronal viabiliy was determined through typical neuronal Na^+^–K^+^ currents. TRPV1 desensitization was evoked by three repetitive 10 s-pulse of 1 μM capsaicin using a gravity-driven perfusion system (2 mL/min; Devesa et al., 2014). BK (1 μM) was applied for 8 min between P2 and P3 capsaicin pulses. Potentiation of TRPV1-mediated currents was calculated as the ratio of P3/P2 current peaks.

### Electrical Properties

Electrical properties of the neuron were determined 2 min after establishing whole cell access using the current-clamp mode. For current-clamp recording, cells were held at 0 pA. Cells were assessed for the presence of spontaneous activity for 1 min and processed to measure electrogenic properties. Both IB4^-^ and IB4^+^ nociceptors were processed for electrogenic properties before BK application. Firing threshold was measured first by injecting a series of 100 ms depolarising current in 10 pA steps from 0 pA to elicit the first AP. To examine the neurons firing properties, a depolarising current of 40 pA (IB4^-^) and 100 pA (IB4^+^) of 100 ms was injected to fire APs. APs were analyzed for the following intrinsic membrane properties: threshold potential (mV), amplitude of AP (mV), duration of AP (ms), overshoot of AP (mV), amplitude of afterhyperpolarization potential (mV). BK (1 μM) was applied to IB4^-^ and IB4^+^ nociceptors for 4 min to observe BK induced spontaneous neuronal excitabilities. RMP was checked before and after BK application. Mean depolarization was calculated by selecting the maximum depolarized voltage observed upon BK application in IB4^-^ and IB4^+^ nociceptors. Nociceptors (IB4^-^ and IB4^+^) were injected with 100 pA for 100 ms and 300 pA for 1 s to measure electrically evoked APs. BK induced changes in electrically evoked APs were measured before and after BK application.

### MEA Measurements

Extracellular recordings were made using multiple electrode planar arrays of 60-electrode thin MEA chips, with 30 μm diameter electrodes and 200 μm inter-electrode spacing with an integrated reference electrode (Multichannel Systems GmbH). The electrical activity of primary sensory neurons was recorded by the MEA1060 System (Multi Channel Systems GmbH^1^), and MC_Rack software version 4.3.0 at a sampling rate of 25 kHz. TRPV1-mediated neuronal firing activity was evoked by three repetitive 15 s-applications of 500 nM capsaicin, using continuous perfusion system (2 mL/min). BK (1 μM) in external solution was perfused between P2 and P3 for 8 min. For experiments on BK receptor agonists, selective BK receptor agonists for BKR1 and BKR2 were applied between P2 and P3 for 8 min. Data were analyzed using MC_RACK spike sorter and Neuroexplorer Software (Nex Technologies). An evoked spike was defined when the amplitude of the neuronal electrical activity overcame a threshold set at -20 μV. The recorded signals were then processed to extract mean spike frequency.

### Data Analysis

All data are expressed as mean ± SEM, with *n* as number of registered cells and *N* as the number of independent experiments. The percentage of sensitized neurons was calculated considering those cells that exhibited a fold potentiation (P3/P2) above 1.1. Statistical analysis was made using the Wilcoxon Rank test or Student’s *t*-test (unpaired or paired), one-way ANOVA or two-way ANOVA followed by Bonferroni’s *post hoc* test as indicated using the GraphPad Prism 5.0 (Graph-Pad). MEA data were analyzed by Bonferroni’s *post hoc* test as paired values through comparison of the responses of each electrode in the 30-s time interval upon stimulation. *p* < 0.05 was considered to be significant for a difference.

## Results

### BK Modulates Electrogenic Properties of Peptidergic and Non-peptidergic Nociceptors

**FIGURE 1:**
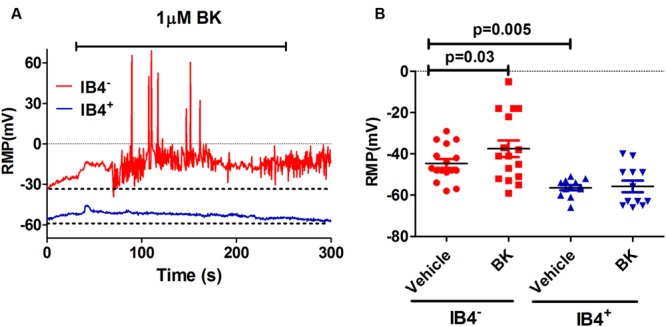
**Effect of BK on modulating excitability of neonatal rat peptidergic IB4^-^ and non-peptidergic IB4^+^ neurons. (A)** Representative of the RMP change in a peptidergic (red) and a non-peptidergic (blue) neuron upon exposure to 1 μM BK. **(B)** Effect of BK on the RMP in from peptidergic and non-peptidergic neuron before and after 1 μM BK application. Each point represents a neuron from three independent cultures. Statistical analysis was performed using the non-parametric Wilconson Rank sum test.

As a preliminary step, we characterized the effect of BK on the electrical properties of peptidergic (IB4^-^) and non-peptidergic (IB4^+^) nociceptors. For this purpose, BK-induced changes in spontaneous neuronal activity, RMP and the properties of electrically evoked APs were investigated in primary cultures of neonatal rat nociceptors. For these experiments, IB4^+^ nociceptors were fluorescently labeled with Alexa-IB4, and nociceptor excitability was evaluated by whole cell patch-clamp. First, neurons were recorded for their RMP for 1 min after seal formation. Thereafter, these nociceptors were perfused with 1 μM BK for 4 min and an electrical response typically began within a minute of the pro-inflammatory agent application (**Figure 1A**). As seen, exposure of IB4^-^ nociceptors to BK produced a depolarization of the RMP that resulted in the firing of APs. In marked contrast, exposure of IB4^+^ neurons to BK did not result in a significant alteration of the RMP nor firing of APs. Quantification of the mean RMP reveals a more depolarized value for IB4^-^ nociceptors (-45 ± 2 mV) than for IB4^+^ neurons (-56 ± 1 mV; **Figure 1B**). Furthermore, despite of the variability inherent in primary nociceptor cultures, BK produced a significant depolarization in the mean RMP value (≈7 mV) of IB4^-^ nociceptors, indicating a direct effect of the pro-algesic agent in the excitability of peptidergic nociceptors.

**FIGURE 2:**
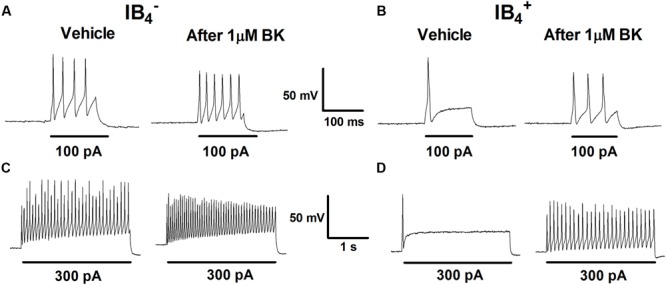
**Effect of BK on evoked responses of IB4^-^ and IB4^+^ soma to depolarizing currents.** Representative traces of APs evoked by injecting a depolarizing current of 100 pA (100 ms) and 300 pA (1 s) delivered to the soma of IB4^-^ **(A,C)** and IB4^+^ **(B,D)** neurons before (Vehicle) and after instillation to 1 μM BK.

Increase in nociceptor excitability or sensitization by inflammatory mediators has been associated with lower threshold requirement to fire APs and to an increased firing frequency in response to depolarizing stimuli (Gold and Traub, 2004). Next, we investigated the impact of BK exposure on electrically evoked APs in nociceptors. First, nociceptors (IB4^-^ and IB4^+^) were examined for evoked excitability in basal conditions upon injecting a depolarizing current pulse of 100 pA for 100 ms or of 300 pA for 1 s (**Figure 2**, vehicle). In both conditions, IB4^-^ nociceptors displayed significantly higher electrically evoked activity than IB4^+^ neurons. Second, 1 μM BK was applied for 4 min and nociceptors were retested for changes in evoked APs. Exposure of IB4^-^ nociceptors to BK depolarized the RMP, and increased the frequency of electrically activated APs by ≥1.5-fold, as evidenced for the two current injections (**Figures 2A,C**). Similarly, exposure of IB4^+^ nociceptors to BK also resulted in an increment in the electrically evoked AP firing, especially when 300 pA were applied (**Figures 2B,D**). Collectively, these findings indicate that peptidergic nociceptors are more excitatable and more reactive to acute BK sensitization than non-peptidergic nociceptors.

### BK-Induced Sensitization of TRPV1 in Nociceptors is Mainly Mediated by BK2R Receptors

**FIGURE 3:**
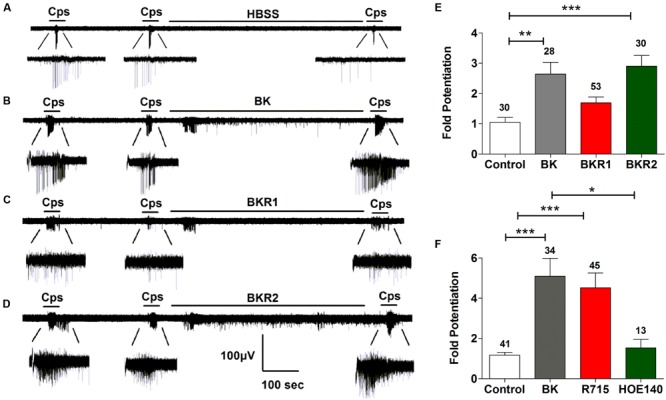
**Sensitization of TRPV1 activity by BK, BKR1 and BKR2 receptor agonists. (A,B)** Representative traces of capsaicin (cps = 500 nM, 15 s) evoked APs in neonatal rat DRG neurons upon applying buffer (Control) or 1 μM of BK between the second (P2) and third (P3) vanilloid pulse, respectively. **(C)** Representative recordings of 1 μM Sar-[D- Phe^8^]-des-Arg^9^-Bradykinin (BKR1), and **(D)** 1 μM BKR2 ([Phe^8^ψ(CH-NH)-Arg^9^]-Bradykinin; BKR2) inducing potentiation of capsaicin evoked neuronal excitability in rat nociceptors. **(E)** BK receptor agonists evoked fold potentiation (ratio P3/P2) of TRPV1 mediated neuronal firing activity. **(F)** BK evoked fold potentiation (ratio P3/P2) of TRPV1 mediated neuronal firing activity in the presence of BK receptor antagonists (1 μM R715 for BKR1, 1 μM HOE140 for BKR2). Data were analyzed as paired values through comparison of the responses of each electrode in the 30 s time interval upon stimulation. Data are expressed as mean ± SEM. The numbers above the bars represents the total number of electrodes that responded. Number of cultures ≥3. Statistical analysis was performed by one-way ANOVA followed by Bonferroni *post hoc* test (^∗^*p* < 0.05, ^∗∗^*p* < 0.01, ^∗∗∗^*p* < 0.001).

We next investigated the potentiating effect of BK on TRPV1 channels. For these experiments, we used the MEA technology to monitor the electrical activity of primary cultures of rat DRG neurons evoked by capsaicin. To register neuronal activity, a continuous protocol was used where two 15 s pulses of 500 nM capsaicin interspersed by a washing period of 3 min were applied, followed by an 8-min incubation with vehicle or 1 μM BK, and finalized by a third (P3) capsaicin pulse (**Figures 3A,B**). As seen, successive capsaicin pulses led to TRPV1 tachyphylaxia as evidenced by the lower AP firing evoked by the second (P2) and third (P3) pulses (**Figure 3A**). Application of BK after the second capsaicin pulse resulted in an increment of neuronal activity directly evoked by BK (**Figure 3B**). Notably, a subsequent capsaicin pulse (P3) produced a significant augment of the vanilloid response as compared with the second pulse (**Figure 3B**), consistent with a BK-induced potentiation of TRPV1 responses. Analysis of the mean spike frequency of each capsaicin pulse and the fold potentiation (estimated as the ratio P3/P2) substantiates this finding, revealing that BK increased the TRPV1 activity by 2.6 ± 0.4 fold (**Figure 3E**).

It has been documented that BK induces nociceptor potentiation mainly through BKR2 receptors, which are expressed constitutively in peptidergic nociceptors (Seabrook et al., 1997; Lee et al., 2002). In contrast, BKR1 receptors expression appears to be induced during inflammation in IB4^+^ non-peptidergic nociceptors (Davis and Perkins, 1994; Vellani et al., 2004; Petho and Reeh, 2012), with limited studies reporting a constitutive expression of BK1R receptors in nociceptors (Wotherspoon and Winter, 2000; Ma, 2001). Thus, the question that raises is which receptor isoform is mediating BK-induced TRPV1 sensitization in nociceptors. To explore the contribution of BKR1 and BKR2 receptors on BK induced TRPV1 sensitization, the BKR1 agonist Sar-[D-Phe^8^]-des-Arg^9^-BK) and the BKR2 agonist ([Phe^8^ψ(CH-NH)-Arg^9^]-BK) were used in an identical experimental paradigm as above. **Figure 3C** illustrates that BKR1 agonist modestly evoked neuronal firing and marginally promoted TRPV1-mediated neuronal spikes. In marked contrast and akin to BK, the BKR2 agonist notably increased nociceptor firing and strongly potentiated TRPV1 induced neuronal spiking (**Figure 3D**). Quantification of the mean spike frequency further substantiates the prominent effect of the BKR2 ligand as compared with that of BKR1. Notably, the fold potentiation of TRPV1-evoked neuronal spikes induced by BKR2 ligands (2.9 ± 0.3) is virtually identical to that evolved by BK (2.6 ± 0.4; **Figure 3E**) implying that BK exerts mainly its action through BKR2. In support of this tenet, no significant increase in the fold potentiation of TRPV1 evoked neuronal spikes was observed in neurons treated with the BKR1 agonist (**Figure 3C**). These results were further corroborated by using specific blockers of both receptor subtypes (**Figure 3F**). Collectively, these findings suggest that BK induces sensitization of TRPV1 mainly through activation of BKR2 in nociceptors.

### BK Sensitized, TRPV1-Mediated Neuronal Firing of Nociceptors is Blocked by DD04107

Recent studies revealed that acute incubation of IB4^-^ nociceptors with BK induced the release of αCGRP in a dose-dependent manner through activation of the Gα_q/11_ signaling pathway (Supowit et al., 2011). Our earlier findings showed that the algogen ATP, that also activates the G_q/11_ signaling pathway, sensitizes TRPV1 in peptidergic nociceptors promoting the exocytosis of LDCVs carrying the receptor (Devesa et al., 2014). Henceforth, we hypothesized that BK may also induce TRPV1 sensitization through the exocytotic recruitment of new TRPV1 channels present in LDCVs.

**FIGURE 4:**
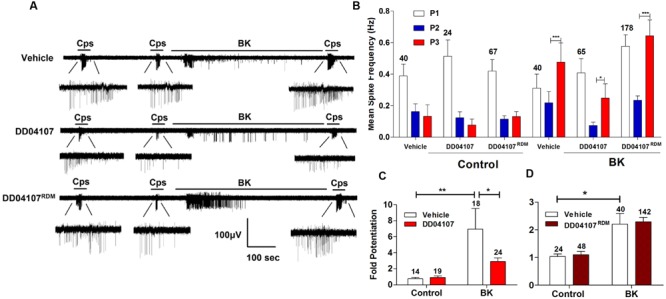
**Peptide DD04107 inhibits BK-sensitized TRPV1-mediated nociceptor excitability. (A)** Representative traces of capsaicin-evoked APs upon applying BK between the second (P2) and third (P3) vanilloid pulses in neonatal rat DRG neurons exposed to vehicle, DD04107 and DD04107^RDM^ peptides. **(B)** Mean spike frequency of capsaicin evoked AP firing under control (buffer) and BK-treated conditions in the presence of vehicle and peptides DD04107 and DD04107^RDM^. **(C,D)** Fold potentiation (ratio P3/P2) of BK-induced potentiation of TRPV1 evoked neuronal firing in the presence of peptide DD04107 and DD04107^RDM^ respectively. Capsaicin (cps = 500 nM, 15 s) and BK (1 μM, 8 min) were used. DD04107 and DD04107^RDM^ (20 μM) were pre-incubated for 1 h at 37°C. Mean spike frequency was calculated from recordings displayed in **(A)**. Data were analyzed as paired values through comparison of the responses of each electrode in the 30 s time interval upon stimulation. Data are expressed as mean ± SEM. The numbers above the bars represents the total number of electrodes that responded. Number of independent cultures ≥3. Statistical analysis was performed by one-way ANOVA repeated measures with Bonferroni’s *post hoc* test and Unpaired Student’s *t*-test (^∗^*p* < 0.05, ^∗∗^*p* < 0.01).

To investigate if BK promotes the membrane mobilization of TRPV1 we evaluated the impact of inhibiting neuronal exocytosis of LDCVs using compound DD04107, a small lipidated peptide that interferes with the formation of the SNARE complex (Camprubí-Robles et al., 2009). For these experiments, we also used the MEA technology to monitor the electrical activity of primary cultures of rat DRG neurons (Devesa et al., 2014) utilizing an identical experimental protocol as above. In control cultures, two successive capsaicin pulses led to TRPV1 tachyphylaxia as evidenced by the lower AP firing evoked by the second (P2) pulse (**Figure 4A**). Application of BK after the second capsaicin pulse to cultures exposed to vehicle resulted in an increment of neuronal activity directly evoked by the kinin (**Figure 4A**, vehicle). A subsequent capsaicin pulse (P3) produced a significant augment of the vanilloid response as compared with the second pulse, consistent with a BK-induced potentiation of TRPV1 responses. Analysis of the mean spike frequency of each capsaicin pulse and the fold potentiation of these experiments (estimated as the ratio P3/P2) substantiates this finding, revealing that BK increased the TRPV1 activity in nociceptors by ≥2.0 fold (**Figures 4B–D**).

We next evaluated the effect of peptide DD04107 on the BK potentiation of capsaicin responses. For these experiments, primary cultures of rat nociceptors were pre-incubated with 20 μM DD04107 for 1 h. As a control, we used 20 μM compound DD04107^RDM^, a peptide with the same amino acid composition but random sequence. Neither peptide affected the capsaicin nor BK responses as illustrated in **Figure 4A** (DD04107 and DD04107^RDM^). As for control conditions, two consecutive pulses of the vanilloid-induced TRPV1 tachyphylaxia. Notably, exposure of nociceptors to 1 μM BK did resulted in a significantly lower potentiation of the response to the third capsaicin pulse in nociceptors incubated with peptide DD04107 as compared with those incubated with vehicle or DD04107^RDM^. This is clearly discerned in the mean spike frequency and in the fold potentiation (**Figures 4B–D**). As illustrated in **Figure 4**, DD04107 notably inhibited the fold potentiation of vanilloid responses evoked by BK (**Figure 4C**). In contrast, DD04107^RDM^ did not affect the sensitizing effect of the kinin (**Figure 4D**). Though we observe a lower potentiation of TRPV1 activity by BK in DD04107-exposed neurons compared to vehicle and DD04107^RDM^-treated nociceptors, we still notice potentiation of TRPV1 evoked excitability upon inhibiting neuronal exocytosis with DD04107 (**Figures 4A,B**). This result is consistent with the tenet that other mechanisms, apart from channel recruitment to the cell surface, are involved in BK-induced TRPV1 sensitization. Taken together, our findings imply that TRPV1 sensitization by BK in nociceptors is partially mediated by the exocytotic recruitment of new TRPV1 channels to the neuronal surface.

### BK Sensitization of TRPV1 in Peptidergic Nociceptors is Sensitive to Blockade of Neuronal Exocytosis

**FIGURE 5:**
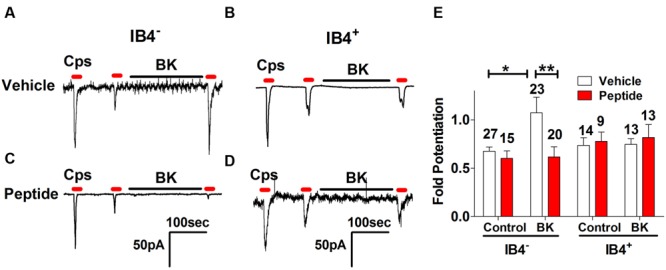
**Effect of non-palmitoylated DD04107 on BK-induced sensitization of TRPV1 channel on rat peptidergic and non-peptidergic DRG neurons. (A,B)** Representative showing the effect of 1 μM BK on the ionic currents elicited by capsaicin (cps = 1 μM, 10 s) in IB4^-^ and IB4^+^ nociceptors, respectively. **(C,D)** Effect of 100 μM non-palmitoylated DD04107 peptide (denoted as Peptide) on the BK-induced potentiation of capsaicin-evoked ionic currents in IB4^-^ and IB4^+^ nociceptors, respectively. **(E)** Fold potentiation (ratio P3/P2) of capsaicin-evoked ionic currents by BK in cultures exposed to vehicle and peptide. Cells were held at -60 mV. Peptide was given through the patch pipette and incubated for 10 min after forming the seal. Data are expressed as mean ± SEM. The numbers above the bars denote the total neurons registered. Number of independent cultures = 4. Statistical analysis was performed using two-way ANOVA with Bonferroni’s post-test (^∗^*p* < 0.05, ^∗∗^*p* < 0.01).

From our previous results, a question that emerges is whether BK-induced exocytotic mobilization of TRPV1 channels to the plasma membrane is a mechanism used by both types of nociceptors or by a specific subtype. Thus, we next examined the effect of peptide DD04107 on BK-induced TRPV1 sensitization in IB4^-^ and IB4^+^ nociceptors. For these experiments, IB4^+^ nociceptors were fluorescently labeled with Alexa-IB4, and TRPV1 channel activity was evaluated by whole cell patch-clamp. **Figure 5A** shows that capsaicin-induced TRPV1 desensitization in IB4^-^ nociceptors was strongly potentiated by 1 μM BK. Notably, BK-promoted TRPV1 sensitization in IB4^-^ sensory neurons was virtually abolished by 100 μM of the non-palmitoylated DD04107 peptide derivative delivered to the neuronal cytosol through the patch pipette. These results are corroborated upon calculating the fold potentiation of TRPV1 currents by taking the ratio of P3 and P2 (P3/P2; **Figure 5E**). No significant change in the fold potentiation was observed under control conditions for vehicle and peptide groups. However, the TRPV1 activity was ≈1.7-fold higher after BK incubation compared to control (**Figure 5E**). Noteworthy, the BK-induced increase in TRPV1 potentiation in IB4^-^ was fully blocked by the non-palmitoylated peptide DD04107 (**Figures 5C,E**), consistent with the tenet that BK sensitizes TRPV1 by promoting its mobilization to the neuronal surface.

At variance with IB4^-^ nociceptors, BK did not induce significant TRPV1 sensitization in IB4^+^ sensory neurons (**Figure 5B**). Consistent with the current recordings, there was no fold potentiation of TRPV1 evoked by BK in IB4^+^ nociceptors (**Figure 5E**) and, consequently, there was no effect of the non-palmitoylated DD04107 peptide (**Figures 5D,E**). Collectively, these results suggest that most of the TRPV1 sensitizing action produced by BK occurs in peptidergic nociceptors with a marginal contribution of the IB4^+^, non-peptidergic population. This is consistent with the finding that BK signals mainly through BKR2.

### Silencing of α-CGRP Abrogates BK Induced Sensitization of TRPV1

**FIGURE 6:**
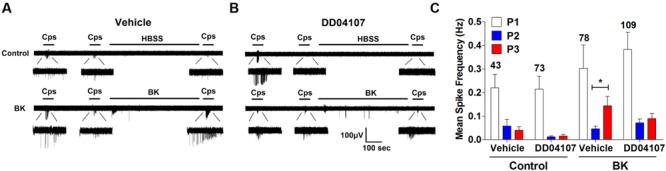
**BK-induced potentiation of TRPV1 evoked neuronal firing in mice nociceptors is sensitive to DD04107. (A)** Representative MEA recordings of capsaicin induced action potentials (APs) and desensitization (*Top*), and potentiation by BK between the second (P2) and third (P3) vanilloid pulse (*Bottom*) in mice nociceptors. **(B)** Representative recordings of BK-induced sensitization of capsaicin-evoked neuronal excitability in mice nociceptors preincubated with 20 μM of DD04107. **(C)** Mean spike frequency of capsaicin induced AP firing in WT nociceptors. Capsaicin (cps = 500 nM, 15 s) and BK (1 μM, 8 min) were used. DD04107 was preincubated for 1 h at 37°C. Mean spike frequency was calculated from recordings displayed in **(A,B)**. Data were analyzed as paired values through comparison of the responses of each electrode in the 30 s time interval upon stimulation Data are expressed as mean ± SEM, *n* = 4 independent cultures. Statistical analysis was performed by one-way ANOVA followed by Bonferroni *post hoc* test. ^∗^*p* < 0.05.

In a previous study, we reported that ATP-evoked TRPV1 potentiation in IB4^-^ peptidergic nociceptors was strictly dependent on the expression of αCGRP in these nociceptors (Devesa et al., 2014). Genetic ablation of αCGRP expression in mice fully abolished inflammatory recruitment of TRPV1 to the neuronal surface. Thus, we next evaluated whether BK-evoked TRPV1 sensitization also required the expression of pro-inflammatory peptides, αCGRP and/or SP, in peptidergic nociceptors. To address this question, we used DRG nociceptors from αCGRP and SP null mice, and monitored the electrical activity evoked with capsaicin using the MEA technology. We first examined the effect of BK sensitizing TRPV1 activity in mice nociceptors in culture. As illustrated in **Figure 6A**, BK potentiated capsaicin responses in mice nociceptors in a slightly lower extend than their counterpart from rat (**Figures 3B and 6A**). This is clearly discerned in the mean spike frequency (**Figures 3E and 6C**). Notice that BK also produced less spikes in mice nociceptors than in rat nociceptors. Furthermore, BK-evoked TRPV1-sensitization was highly sensitive to the inhibitory action of DD04107 (**Figures 6B,C**), indicating a similar sensitizing mechanism to that reported for rat nociceptors, namely recruitment of new channels to the neuronal surface.

**FIGURE 7:**
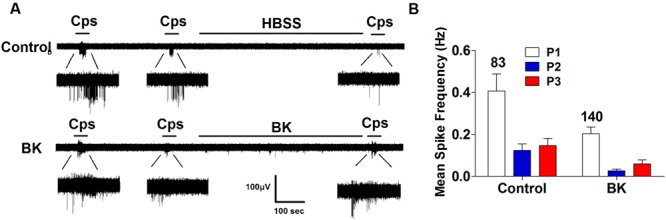
**BK-induced TRPV1 sensitization in αCGRP^-/-^ nociceptors. (A)** Representative traces of capsaicin-evoked APs in primary nociceptor cultures of DRGs from αCGRP^-/-^ mice upon exposure to buffer (*Top*) or BK (*Bottom*) between the second (P2) and third (P3) vanilloid pulse. **(B)** Mean spike frequency of capsaicin-evoked AP firing under control and BK treated conditions. Capsaicin (cps = 500 nM, 15 s) and BK (1 μM, 8 min) were used. Mean spike frequency was calculated from recordings displayed in **(A)**. Data were analyzed as paired values through comparison of the responses of each electrode in the 30 s time interval upon stimulation. Data are expressed as mean ± SEM. The numbers above the bars represents the total number of electrodes that responded. Number of independent cultures ≥4. Statistical analysis was performed by one way ANOVA repeated measures with Bonferroni’s *post hoc* test.

**FIGURE 8:**
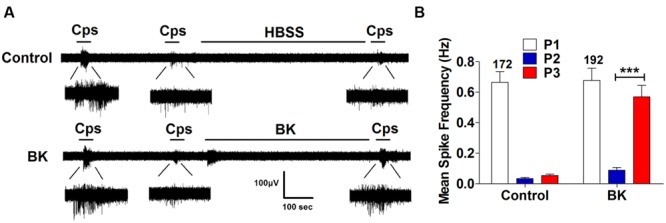
**BK-induced TRPV1 sensitization in Tac1^-/-^ nociceptors. (A)** Representative traces of capsaicin-evoked APs in primary nociceptor cultures of DRGs from Tac1^-/-^ mice upon exposure to buffer (*Top*) or BK (*Bottom*) between the second (P2) and third (P3) vanilloid pulse. **(B)** Mean spike frequency of capsaicin-evoked AP firing under control and BK treated conditions. Capsaicin (cps = 500 nM, 15 s) and BK (1 μM, 8 min) were used. Mean spike frequency was calculated from recordings displayed in **(A)**. Data were analyzed as paired values through comparison of the responses of each electrode in the 30 s time interval upon stimulation. Data are expressed as mean ± SEM. The numbers above the bars represents the total number of electrodes that responded. Number of independent cultures ≥3. Statistical analysis was performed by one-way ANOVA repeated measures with Bonferroni’s *post hoc* test (^∗∗∗^*p* < 0.001).

Next, we performed the same measurements in nociceptors from αCGRP (αCGRP^-/-^, **Figure 7**) and SP (Tac^-/-^, **Figure 8**) null mice. In sensory neurons from αCGRP^-/-^ mice, BK was unable to potentiate TRPV1-evoked neuronal excitability (**Figures 7A,B**), similar to the blockade exerted with DD04107. In contrast, BK evoked strong TRPV1 sensitization in nociceptors from Tac^-/-^ mice (**Figure 8**). These results clearly imply that, akin to ATP, BK-promoted TRPV1 sensitization in IB4^-^ peptidergic nociceptors was dependent on the expression of αCGRP, and further imply that a population of TRPV1 is trafficked to the neuronal terminals in LDCVs loaded with αCGRP. Thus, pro-inflammatory mediators sensitize nociceptors by the concomitant release of αCGRP and the membrane recruitment of TRPV1 channels. This finding further corroborates that inflammatory sensitization of TRPV1 in peptidergic nociceptors involves the exocytotic delivery of new channels trafficked by LDCVs to the neuronal surface.

## Discussion

Algesic sensitization of nociceptor peripheral terminals leading to enhanced excitability is a major component of pain transduction. In physiological conditions, peripheral nociceptor terminals are very silent, and only activated when a noxious stimulus is perceived, therefore acting as a protective mechanism to preserve tissue integrity. In contrast, upon tissue damage or inflammation, a plethora of algogen factors are released from damaged nociceptors and surrounding immune cells that act on nociceptor receptors, primarily augmenting nociceptor excitability (Gold and Traub, 2004), i.e., an increase in the firing of APs that leads to pain (Mamet et al., 2002). A pivotal action of released algogens is the potentiation of TRPV1 channel activity, an AP generator in nociceptors, by favoring its gating and augmenting its membrane expression (Morenilla-Palao et al., 2004; Srinivasan et al., 2008; Camprubí-Robles et al., 2009). We previously reported that membrane mobilization of TRPV1 channels to the neuronal surface by pro-algesic agents is a mechanism involved in the *in vitro* and *in vivo* sensitization of nociceptors as its blockade by compound DD04107, an inhibitor of neuronal exocytosis, attenuates receptor sensitization and results in antinociception (Camprubí-Robles et al., 2009; Ponsati et al., 2012). Similarly, Botulinum neurotoxin A is also an effective anti-nociceptive drug widely used to treat several painful human syndromes (Cui et al., 2004; Shimizu et al., 2012; Kim et al., 2015; Sidaway, 2015), implying that algesic mobilization of TRPV1 may be a general mechanism for nociceptor sensitization. However, we found that ATP-induced TRPV1 sensitization involved membrane mobilization of new channels only in peptidergic nociceptors (Devesa et al., 2014). These findings imply that inflammation-induced surface recruitment of TRPV1 may be a sensitizing mechanism used by peptidergic nociceptors that cargo TRPV1 in αCGRP-loaded LDCVs.

We have further tested this hypothesis and investigated the molecular sensitizing mechanism of BK, a kinin that acts as a strong pro-algesic agent (Walker et al., 1995). The salient contributions of this study are that: (i) BK directly triggers electrical activity and strongly sensitizes peptidergic nociceptors, while exhibits a moderate effect on non-peptidergic neurons; (ii) acute BK nociceptor sensitization is mainly through BKR2, which are highly expressed in peptidergic nociceptors. (iii) BK potentiates TRPV1 activity in peptidergic nociceptors by a mechanism that partially involves the mobilization of channels trafficked in αCGRP-loaded LDCVs. And, (iv) BK sensitization of TRPV1 activity in peptidergic nociceptors is strictly dependent of αCGRP expression, and it is abrogated by blockade of Ca^2+^-activated, SNARE-mediated neuronal exocytosis with DD04107. Taken together, these findings indicate that inflammatory sensitization of TRPV1 in peptidergic nociceptors is significantly mediated by the mobilization of new channels to the neuronal surface. Furthermore, they substantiate that acute peripheral inflammatory sensitization induced by BK and ATP is largely facilitated by peptidergic nociceptors with a moderate contribution of the non-peptidergic subpopulation. In support of this tenet, we also observed that sensitization of nociceptors with a mixture of BK and ATP was notably attenuated by blocking neuronal exocytosis with DD04107 (data not shown). These results corroborate our previously published ATP experiments, suggesting that both ATP and BK activate Gα_q/11_ signaling protein and induce TRPV1 exocytosis in peptidergic nociceptors. Recently, *in vivo* studies have been performed using Gα_q_^-/-^, Gα_11_^-/-^, and Gα_q/11_^-/-^ knockout mice models to understand the role of Gα_q/11_ signaling protein on nociceptor sensitization induced by algogens. The studies showed that both ATP and BK induced thermal hyperalgesia was preserved in Gα_11_^-/-^ deficient mice, whereas, it was completely abolished in Gα_q_^-/-^ and Gα_q/11_^-/-^ deficient mice. This corroborates that the major common signaling mechanism involved in ATP and BK induced nociceptor sensitization is mediated through Gα_q_ protein and provokes thermal hyperalgesia, where TRPV1 could play a significant role (Wirotanseng et al., 2013).

It is notorious the differential sensitizing effect of acute BK on peptidergic and non-peptidergic nociceptors. In our experimental conditions, BK mainly excited the peptidergic nociceptor population. Indeed, the non-peptidergic nociceptors were rather resistant to BK sensitization, as they were more silent and required stronger stimulation to trigger APs in agreement with their more hyperpolarized RMP. A plausible explanation for this difference may be the differential expression of BK receptor types in both populations of sensory neurons. In support of this hypothesis, a BKR1 agonist did not significantly sensitized our rat nociceptor primary cultures. In contrast, an agonist of the BKR2 fully mimicked the extent of BK sensitization. Furthermore, blockade of BKR2 fully inhibited BK-induced TRPV1 sensitivity while BKR1 antagonists were ineffective. This observation is in agreement with evidence showing that BKR2 are constitutively expressed in nociceptors, while BKR1 expression is induced, especially in chronic pain conditions (Vellani et al., 2004). Accordingly, our results indicate that acutely instilled BK potentiates TRPV1 activity in peptidergic nociceptors primarily through the BKR2 by inducing the exocytosis of αCGRP LDCVs that cargo the thermoTRP channel. Nonetheless, TRPV1 sensitization also involves modulation of channel gating as BK potentiation was also abrogated by PKC blockade (data not shown). Furthermore, our finding substantiate the tenet that acutely BK sensitization is promoted by BKR2 signaling, while BKR1 may be involved in chronic pain. Additional experimentation is required to clearly define the molecular mechanism involved in acute BK sensitization of nociceptors.

We previously reported that acute exposure of primary cultures of nociceptors to BK potentiated TRPV1 responses by a mechanism that did not involve membrane mobilization of the thermoTRP channel (Camprubí-Robles et al., 2009), contrasting with the findings described here. These apparent contradictory results likely arise from the different experimental conditions used in both studies. Firstly, the intensity of the BK stimulus here was 10-fold higher (1.0 μM BK) than that used by Camprubí-Robles et al. (2009; 0.1 μM BK), which resulted in a stronger sensitization of the cultured nociceptors. Secondly, here we have evaluated the effect on the nociceptor excitability, while in Camprubí-Robles et al. (2009) capsaicin-evoked Ca^2+^ fluxes were determined, which could involve TRPV1 and other Ca^2+^-permeable channels activated by membrane depolarization. Thirdly, here we have differentially evaluated the effect in peptidergic and non-peptidergic nociceptors, while in Camprubí-Robles et al. (2009) the response of all nociceptor types was averaged. Independent of the differences between both studies, here we have shown that BK strongly potentiates TRPV1 channel activity in rodent peptidergic nociceptors in culture resulting in robust neuronal sensitization by partially augmenting the recruitment of new channels to the plasma membrane through exocytosis of vesicular channels.

We have also shown that inhibition of Ca^2+^-dependent, SNARE-mediated exocytosis of TRPV1 channels is a pharmacological strategy to reduce the over-excitability induced by BK. Indeed, peptide DD04107 remarkably reduced the BK-induced TRPV1 potentiation in peptidergic nociceptors, which resulted in a reduction of the capsaicin-evoked firing of APs. As expected the reduction of BK-induced TRPV1 sensitization in nociceptors by DD04107 was not complete, consistent with reports indicating that the kinin also sensitizes TRPV1 channels by promoting the PKC phosphorylation of the channel (Sugiura et al., 2002; Wang et al., 2015), and the production of endogenous TRPV1 agonists via phospholipase A_2_-lipoxygenase pathway (Shin et al., 2002). Nevertheless, our results lend support to the outstanding *in vivo* anti-nociceptive activity of peptide DD04107 (Ponsati et al., 2012), and indicates that the concomitant modulation of algesic-induced αCGRP release and membrane mobilization of TRPV1 channels in peptidergic nociceptors is a promising therapeutic strategy for pain intervention. In support of this tenet, a recent report has shown that TNFα induces the co-trafficking of TRPV1 and TRPA1 in LCDVs to the nociceptor membrane and that botulinum neurotoxins A and C1 inhibited the TNFα elevated delivery (Meng et al., 2016).

## Conclusion

Our study reveals that distinct signaling mechanisms are involved in TRPV1 sensitization induced by BK in peptidergic and non-peptidergic nociceptors. Furthermore, substantiate the notion that, similar to ATP, BK induces exocytotic membrane mobilization of TRPV1 trafficked by αCGRP-containing LDCVs in peptidergic nociceptors. Furthermore, these findings lend support to the tenet that algesic recruitment of thermoTRPs channels and the concurrent release of pro-inflammatory peptides provides a synergistic mechanism that ensures rapid modulation of pain peripheral signaling (Devesa et al., 2014; Meng et al., 2016).

## Author Contributions

SM performed research; ID and AF-M designed research; J-PC contributed new reagents/analytical tools; SM and AF-M analyzed data; and AF-M wrote the paper.

## Conflict of Interest Statement

AF-M is an inventor of a patent application protecting the algesic activity of DD04107. WO 2010/009892 A2. The remaining authors declare that the research was conducted in the absence of any commercial or financial relationships that could be construed as a potential conflict of interest.

The reviewer JB and handling Editor declared their shared affiliation, and the handling Editor states that the process nevertheless met the standards of a fair and objective review.

## Footnotes

1 http://www.multichannelsystems.com

## Abbreviations

AP: action potential
BK: bradykinin
αCGRP: α calcitonin gene-related peptide
cps: capsaicin
IB4: isolectin B4
LDCV: large-dense core vesicle
MEA: multielectrode array
RMP: resting membrane potential
SP: substance P
Tac: tachykinin
TRPV1: transient receptor potential vanilloid I
